# Gastroenteritis Aggressive Versus Slow Treatment For Rehydration (GASTRO). A pilot rehydration study for severe dehydration: WHO plan C versus slower rehydration

**DOI:** 10.12688/wellcomeopenres.12261.1

**Published:** 2017-08-10

**Authors:** Kirsty A. Houston, Jack G. Gibb, Ayub Mpoya, Nchafatso Obonyo, Peter Olupot-Olupot, Margeret Nakuya, Jennifer A Evans, Elizabeth C George, Diana M Gibb, Kathryn Maitland

**Affiliations:** 1 KEMRI-Wellcome Trust Research Programme, Kilifi, Kenya; 2 Department of Paediatrics, Faculty of Medicine, St Mary’s Campus, Norfolk Place, Imperial College London, London, UK; 3 Mbale Regional Referral Hospital, Mbale, Uganda; 4 Mbale Clinical Research Institute, Mbale, Uganda; 5 Soroti Regional Referral Hospital, Soroti, Uganda; 6 Department of Paediatrics , University Hospital of Wales, Cardiff, UK; 7 Medical Research Council (MRC) Clinical Trials Unit, University College London, London, UK

**Keywords:** acute gastroenteritis, children, intravenous fluids, WHO, dehydration

## Abstract

Background: The World Health Organization (WHO) rehydration management guidelines (Plan C) for children with acute gastroenteritis (AGE) and severe dehydration are widely practiced in resource-poor settings, yet have never been formally evaluated in a clinical trial. A recent audit of outcome of AGE at Kilifi County Hospital, Kenya noted that 10% of children required high dependency care (20% mortality) and a number developed fluid-related complications. The fluid resuscitation trial, FEAST, conducted in African children with severe febrile illness, demonstrated higher mortality with fluid bolus therapy and raised concerns regarding the safety of rapid intravenous rehydration therapy. Those findings warrant a detailed physiological study of children’s responses to rehydration therapy incorporating quantification of myocardial performance and haemodynamic changes.

Methods: GASTRO is a multi-centre, unblinded Phase II randomised controlled trial of 120 children aged 2 months to 12 years admitted to hospital with severe dehydration secondary to AGE. Children with severe malnutrition, chronic diarrhoea and congenital/rheumatic heart disease are excluded. Children will be enrolled over 18 months in 3 centres in Kenya and Uganda and followed until 7 days post-discharge. The trial will randomise children 1:1 to standard rapid rehydration using Ringers Lactate  (WHO plan ‘C’ – 100mls/kg over 3-6 hours according to age, plus additional 0.9% saline boluses for children presenting in shock) or to a slower rehydration regimen (100mls/kg given over 8 hours and without the addition of fluid boluses). Enrolment started in November 2016 and is on-going. Primary outcome is frequency of adverse events, particularly related to cardiovascular compromise and neurological sequelae.  Secondary outcomes focus on clinical, biochemical, and physiological measures related to assessment of severity of dehydration, and response to treatment by intravenous rehydration.

Discussion: Results from this pilot will contribute to generating robust definitions of outcomes (in particular for non-mortality endpoints) for a larger Phase III trial.

## Introduction

Worldwide, an estimated 2.5 billion cases of acute gastroenteritis (AGE) occur annually in children under 5 years. In these children, gastroenteritis is the second biggest cause of mortality (after acute respiratory illnesses) with the vast majority occurring in low resource settings such as sub-Saharan Africa ^1^. A large case-control study called the Global Enteric Multicentre study (GEMS) carried out in Africa and Asia showed that patients with moderate or severe gastroenteritis are 8.5 times more likely to die than non-gastroenteritis controls ^2, 3^. A third of the fatalities occurred < 7days following hospitalisation, indicating that current management strategies may not be working in practice.

WHO has produced guidance on management of dehydration in children with diarrhoea ^4^. Oral Rehydration Solution (ORS) is recommended for children with diarrhoea, with some dehydration defined as two or more of the following: restlessness/irritability, sunken eyes, drinks eagerly/thirsty and/or skin pinch goes back slowly (Plan B). Intravenous fluids are recommended for resuscitation of children with severe dehydration, using 100mls/kg of Ringers Lactate or 0.9% Saline. Severe dehydration is defined as two or more of the following features: sunken eyes, skin pinch goes back very slowly ≥2seconds, lethargy, and/or inability to drink, (under the protocol called ‘Plan ‘C’). 100mls/kg, is the approximate volume estimated to have been lost in children with 10% dehydration and is recommended to be given over 3 hours (or 6 hours in children < 1 year). There are two steps to this regime:

Step 1: 30mls/kg over 30mins (or 1 hour if <1yr);

Step 2: 70mls/kg over 2.5 hours (or 5 hours if <1year).

For children presenting with shock (defined as presence of all three of: weak and fast pulse, temperature gradient and capillary refilling time >3seconds) WHO recommends initial fluid boluses given for shock (i.e. up to 3 boluses of 20mls/kg of normal (0.9%) saline given as rapidly as possible) followed directly by step 2, i.e. 90-130mls/kg. These management guidelines are widely practiced in resource-poor settings, despite never having been formally tested in a clinical trial. In a review of the evidence for WHO management guidelines in 2012, with regard to shock and rehydration management, the review focused principally on type of fluid for resuscitation but did not consider the rate or volume ^5^.

### Evidence from trials and audits

In the Phase III FEAST randomised controlled trial, fluid bolus therapy was compared with maintenance fluids in children with signs of shock and febrile illness (but NOT in children with AGE, severe malnutrition or other causes of fluid loss) ^6^. The results showed a 3.3% higher absolute mortality in children randomised to fluid bolus therapy than in the maintenance only control arm (4mls/kg/hour). A terminal clinical events sub-analysis (conducted blind to randomised arm allocation by the endpoint review committee) suggested the excess mortality in the FEAST trial was significantly attributable to cardiogenic collapse (rather than fluid overload) with no difference in the neurologic or respiratory events by study arm ^7^. We had proposed that the vasoconstrictive response in shock may be an adaptive response to severe infection, protecting blood flow to vital organs and that the rapid correction of shock by bolus therapy may be deleterious ^6^. Alternatively, cardiovascular collapse may occur as a result of right atrial stretch following fluid boluses therapy, and could be predicted by a raised ANP (atrial natriuretic peptide) and subsequent urinary sodium excretion ^8^. This has raised concerns regarding the safety of rapid intravenous rehydration therapy in resource-poor settings and also in other common illnesses requiring aggressive fluid such as acute gastroenteritis. In resource-poor settings, where there is no access to mechanical ventilation, the effect of fluid bolus administration could be studied using non-invasive echocardiography imaging to quantify myocardial performance indices and haemodynamic response to rehydration therapy; serial assessment of cardiac biomarkers and urinary sodium might also be valuable.

A systematic review of intravenous rehydration worldwide found only 3 trials (n=464) comparing rates of rehydration; none were conducted in resource-poor settings ^9^. There were no deaths in any trial but a pooled analysis suggested longer time-to-discharge and higher readmission rates in the rapid rehydration arms. A recent audit of outcomes from AGE at Kilifi County Hospital, Kenya found that 10% of children with gastroenteritis managed on the WHO Plan C protocol required high dependency care. Mortality was up to 20% and a number ****developed fluid-related complications including cardiovascular collapse or neurological complications (status epilepticus or coma). Emerging data on outcomes from AGE across a network of hospitals in Kenya, including 1211 children with severe dehydration who were managed with the current WHO plan C, found an in-hospital mortality of 12% in those with severe dehydration; mortality increased to 31% in children with additional complications of shock ^10^.

Given the information provided by the FEAST trial and the high early mortality for AGE (noted in Kilifi and globally in the GEMS study ^2^), we believe it is now crucial to further investigate the impact of different rates of rehydration on physiological outcomes in different groups of patients. The data generated by this pilot Phase II trial focuses on safety and surrogates of efficacy of two strategies for rehydration, with the potential to inform the design of a future definitive multi-site Phase III trial in children presenting with severe dehydration secondary to AGE.

## Protocol

### Justification for the study

This pilot study will be the first step in evaluating the current evidence-base for the WHO rehydration protocols that are widely used in severely dehydrated children with gastroenteritis. We have chosen to evaluate the WHO Plan C, since the evidence base for both WHO Plan ‘C’ and country specific protocols such as the Kenya Paediatric protocols, have been judged to be of very low quality ^5^, relying solely upon expert opinion that is not based upon evidence from clinical trials. GASTRO was designed to evaluate the safety of the current recommendation and a slower rehydration strategy that also omits initial shock correction. In addition, we aimed to determine the most appropriate secondary outcomes to be included in the design of a future Phase III trial.

### Null hypothesis

Slower rehydration is equally effective at correcting dehydration but is associated with fewer fluid related adverse effects.

## Objectives

### General objectives

To compare the current standard WHO plan ‘C’ rehydration protocol with a strategy that aims to deliver a slower rehydration regimen (which is also simpler to administer) using the same total volume (100ml/kg of Ringers Lactate) over 8 hours, irrespective of age.

### Specific objectives

a)  To compare serious adverse events (SAEs) and adverse events, particularly related to cardiovascular compromise, respiratory, and neurological sequelae by randomised arm.

b)  To compare clinical, biochemical, and physiological data by randomised arm, on:

i. Initial assessment of dehydration
ii. Response to IV rehydration.

c)  To inform robust definitions of primary and secondary outcomes for a larger phase III trial

### Design and methodology

The study will be conducted in Kilifi County Hospital, Kenya, Mbale Regional Referral Hospital (MRRH), Uganda and Soroti Regional Referral Hospital (SRRH), Uganda. GASTRO study progress at the time of writing is detailed in the ‘Study progress’ section below.

### Study design

A multi-centre, open-label pilot phase II trial comparing slow versus standard (fast) intravenous rehydration of children admitted to hospital with gastroenteritis and severe dehydration

### Study populations

***Inclusion criteria:*** Children aged 60 days to 12 years with gastroenteritis (> 3 loose stools/day) and signs of severe dehydration (as per WHO definition – unable to drink or AVPU <A, with sunken eyes and reduced skin pinch (<2 seconds) and an inability to take or retain oral fluids), with or without shock. Shock will be defined according to the recent 2016 WHO ETAT criteria as all of the following: cold peripheries with a weak and fast pulse (rate not specified) and a capillary refill time >3 seconds ^4^.

***Exclusion criteria:*** These include: Severe malnutrition (kwashiorkor or MUAC <11.5cm); Diarrhoea lasting more than 14-days; known congenital or rheumatic heart disease; refusal of consent by parent/guardian.

### Sample size determination

We did not calculate a formal sample size. We aim to study 120 children (60 in each arm); this will provide sufficient pilot data (clinical and physiological) on the main outcomes given the timeframe of the study. We have used similar sample sizes in the past in fluid resuscitation studies (comparing rates and different types of fluids that informed the design of FEAST) ^11^. At least half these children will be admitted in Uganda (60), however recruitment is competitive and will finish once 120 children have been enrolled. We will aim for minimum of 40 children to have physiological data collected (Bioelectrical impedance, CytoCam and Echocardiography).

### Study methods and procedures

All children admitted with an acute history of gastroenteritis will be screened for study inclusion by the paediatric triage/admission team. Children enrolled into GASTRO will be transferred to the high dependency ward, Kilifi, Kenya or designated study beds on MRRH Children’s Ward or SRRH Children’s Ward, Eastern Uganda following consent.

### Consent

Once eligibility has been confirmed, authorized trial staff will approach parents/guardians to invite their child to take part in the trial. An information sheet will be provided to the parent/guardian in their usual language, for example Swahili or Giriama, containing details of the GASTRO trial. The sheet will be read aloud by a member of the fieldworker or nursing team to those who are unable to read. The trial authorised doctor/nurse will check that the information has been fully understood, and parents/guardians will be encouraged to ask questions they may have about their child’s participation in GASTRO. Where possible, prospective written informed consent will be sought from parents/guardians who will then be asked to sign the Consent Form. If parents/guardians are unable to sign, a thumbprint will be taken in lieu of a signature. A copy of the Consent Form will be given to the parent/guardian, the original placed in the patient’s medical notes, and a copy kept in the Investigator Site File (see Supplementary File 1).

### Randomisation

Randomisation is stratified by clinical centre. Children will be randomly assigned 1:1 to fast (WHO standard Plan C plus additional boluses for treatment of shock) or slower rehydration without fluid boluses) in an open-label trial. The treatment allocation (Plan C versus slow rehydration) will be kept in numbered, sealed opaque envelopes. The cards are numbered consecutively and are opened in numerical order. An independent statistician at the KEMRI Wellcome Trust Programme will prepare the randomisation list and envelopes, and the list will not be available to investigators.

### Clinical management and monitoring

Following consent and randomisation, the patients will be commenced on the IV rehydration protocol as detailed in Table 1. Clinical and haemodynamic responses will be monitored at 1, 2, 4, 8, 12 hours, then daily until discharge. At each clinical assessment children will be assessed for pre-specified serious adverse events of interest (new onset seizures or worsening conscious level, signs of pulmonary oedema and signs of cardiac failure after the initiation of intravenous rehydration). Routine blood samples will be collected at admission and at 24 hours. At 8 hours an additional 4.5ml blood sample will be collected for biochemistry (blood gas, lactate, electrolytes, and renal function). We will collect samples of urine at admission, 8 hours, and 24 hours and on day 7-post discharge to measure urinary electrolytes (and osmolality where lab facilities enable). All children will have a blood sample collected for analysis of cardiac biomarkers (cardiac troponin I, cTnI; A-type and B-type natriuretic peptides, ANP & BNP) at admission, 8, and 24 hours. All children will have a routine HIV test at hospital admission, in accordance with national guidelines.

**Table 1.** Trial treatment arms. Shock is defined as presence of all three of the following: Weak and fast pulse, temperature gradient and prolonged capillary refill time >3seconds ^4^.

	WHO Plan C		Slow Arm	
	No shock	With Shock	No Shock	With Shock
Aged <12 months	**Step 1** 30mls/kg over 1hr **Step 2** 70mls/Kg over 5hrs	**Resuscitate:** Up to 3 x 20ml/kg bolus **then to Step 2** 70mls/kg over 5hrs	100mls/kg over 8 hours	100mls/kg over 8 hours with No additional boluses
Aged >12 months	**Step 1** 30mls/kg over 30min **Step 2** 70mls/kg over 2.5 hrs	**Resuscitate:** Up to 3 x 20ml/Kg bolus **then to Step 2** 70mls/kg over 2.5hrs	100mls/kg over 8 hours	100mls/kg over 8 hours No additional boluses

Serious adverse events (SAE) will be reported as an adverse event on a standardised SAE form and will be sent to the Clinical Trials Facility, Kilifi, Kenya and to the local ethics board within 2 days. For SAE’s occurring in Kilifi, the local ethics board will be Kenya Medical Research Institute’s Scientific and ethical review unit (SERU), and in Mbale and Soroti this will be Mbale Regional Hospital Institutional review committee (MRHIRC). Independent monitors also monitor all events against source documents. In addition, an independent clinician will remove all references to randomised arm prior to review by the Endpoint Review Committee (ERC). The ERC will have access to clinical narratives, bedside vital observations, serial laboratory and bedside blood tests and concomitant treatments. They will be adjudicated (blind to randomised arm) on whether fatal and non-fatal events could be related to rate of the fluid rehydration, and the main cause of death. Independent endpoint review (and blinding) is essential to improve the robustness of the event review and minimises bias in an open trial. The GASTRO study also has a data and safety monitoring board (DSMB) that will oversee any SAE assessments (see DSMB charter, Supplementary File 2).

### Clinical management

Following correction of dehydration (based on a review of WHO clinical signs and observations), children will be assessed for their ability to take oral rehydration or feeds. Children who are able to take and retain oral fluids/feeds and who are in neutral or marginally positive fluid balance (both input and output will be measured as accurately as possible) will be considered as satisfactorily rehydrated. All children will be offered oral rehydration fluids alongside their IV rehydration regimen. Each child will have an input-output monitoring chart i.e. including urinary catheter volumes and diaper weights, to document the volumes that children in both arms are drinking and retaining, as well as defining clinical end-points that will be used to guide when to stop IV fluids. For the purposes of this study, each child will aim to complete their allocated IV fluid hydration regimen. In order to achieve accurate fluid balance calculations we have included use of urinary catheters in the trial, which is specifically detailed in the patient information sheet.

In the case of a child who develops clinical signs and fluid overload, the management plan is to stop IV fluids; if there are clear signs of pulmonary oedema, IV frusemide (1–2mg/Kg) and supplemental oxygen will be administered, the child will be closely monitored with hourly observations until stable and further fluid management to be administered orally (or via NGT if the child is unable to take fluids orally). If, after the initial rehydration regimen is completed, there are on-going significant GI fluid losses, we will repeat the fluid regime will be repeated as per their randomised arm; after this, if losses continue, fluid management will be personalized to take account of input/output.

Children will be followed up on Day 7 post discharge. A clinician will review them and they will have their weight and observations recorded. All children will have one further blood sample taken (blood gas, lactate, electrolytes, renal function and cardiac biomarkers). We will also collect one further urine sample for urinary electrolytes. Where children have had BIA and/or Cytocam analysis, these children will have repeat bioelectrical impedance analysis (BIA) and/or CytoCam assessment. These assessments of hydration (in children without any on-going losses or intercurrent illness) serve to validate inpatient assessments (see below).

### Outcome measures

**Primary**

- Frequency of fluid related adverse events

**Secondary**

- Correction of dehydration
- Dysnatraemia at 8 hours
- Time to tolerate oral fluids/feeds
- Time to discharge
- Readmission rate (within 1 week post discharge)

### Sub-studies

***Bioelectrical impedance analysis.*** All children will have a daily weight and fluid balance calculated until discharge (performed at the same time each day) and at follow up. A selection of 40 children admitted to KCH and Mbale will have BIA performed at admission, 8 hours and 24hours. BIA is a non-invasive measurement of cellular health (including hydration) and hydration status of body compartments is widely used to predict prognosis independent of age, weight, and body fat content. Clinical applications of BIA as a prognostic indicator have been well demonstrated in patients with malnutrition, liver cirrhosis, HIV and sepsis. Measurement of BIA alongside urinary electrolytes will allow us to validate the use of BIA in children with acute gastroenteritis. BIA measurement involves application of two electrodes to the child’s wrist and ankle while they are lying still. A small volume current is passed through the electrodes and measures various parameters of body composition including total body water. The procedure does not involve any pain or discomfort to the child and lasts approximately 3–5 minutes.

### Myocardial performance

Up to 40 children admitted to KCH and MRRH will undergo echocardiography. We aim to study in greater depth myocardial, haemodynamic responses and microvascular perfusion to the fluid strategies using non-invasive ultrasonography and echocardiography (Vivid q N BT12 Echo Ultrasound Machine, KEMRI-WTRP). Echocardiography data collection will be standardized; 80 frames/sec in the parasternal, apical and subcostal windows at admission (0hr), 1, 4, 8 and 24 hours. Two-dimensional grayscale three-beat ECG gated loops will be obtained in the apical long axis, apical 4-chamber, apical 2-chamber, parasternal short axis (at basal, mid-papillary and apical levels), parasternal long axis and subcostal views and stored for retrospective/offline analysis of ventricular systolic and diastolic function as well as myocardial strain and torsion. Standard colour Doppler imaging with pulsed and continuous waves will be used to quantify the maximal flow velocities, pressure gradients and regurgitation (if any) across the aortic, pulmonic, mitral and tricuspid valves. In addition, in Kilifi, for a selection of children we will examine microvascular perfusion using an incident dark-field CytoCam machine (Braedius, KEMRI-WTRP).

### Data management and statistical analysis

All clinical and laboratory data will be recorded in the CRF and stored with a unique serial number identifier. Data will be entered onto Open Clinica. All data will be anonymised prior to presentation or publication of any results. As per the statistical analysis plan (see Supplementary File 3), qualitatively clinical data will be summarised by arm, using means and medians where appropriate for continuous data. Primary safety analyses will compare the proportion of children with a pre-specified significant adverse event at 48hours in the Plan C and GASTRO slow arms using an unadjusted chi-squared test. Secondary analyses of efficacy will use Kaplan Meier and log rank tests to compare time to correction of dehydration, time to pass urine and time to tolerate oral fluids. Chi-squared tests will compare incidence of dysnatraemia and readmission rates between the two groups. Data on all measures of cardiac structure and function will be assessed for normality, and means (and standard deviations) or medians (and interquartile ranges) presented as appropriate. Cardiac function during fluid management at presentation, at key time points (admission, 1, 4, 8 and 24 hours), during any deterioration, and at follow up will be described and compared between the two arms in terms of the following: (1) the prevalence of clinical features of heart failure (classically described by gallop rhythm, bi-basal lung crepitations, raised jugular venous pressure, enlarged liver); (2) echocardiographic measures of systolic, diastolic, and global cardiac function, left ventricular strain (radial, longitudinal and circumferential), torsion, stroke volume, cardiac output and inferior vena cava collapsibility index; (3) the prevalence of any ECG abnormalities (such as prolonged QT interval or arrhythmias); (4) biochemical markers of cardiac dysfunction.

### Ethical statement

Ethical and regulatory approval has been granted by Imperial College Research Ethics Committee (ICREC, 16IC3388, initial approval: 18/08/2016), the sponsor of the study; KEMRI Scientific & Ethics Review Unit (SERU, 053/3299, initial approval: 16/08/2016) and Poisons and Pharmaceuticals board, Kenya (PPB, ECCT/16/09/01/2016(156), initial approval: 20/09/2016) and from Mbale REC (initial approval: 28/11/16) and Uganda (UNCST HS2163, initial approval: 16/01/17). GASTRO study has been registered on ISRCTN (registry identifier: 67518332).

### Safety

The study will be performed in patients who may potentially benefit from the treatment. The risks of cannula insertion and blood drawing include pain, infection at the site of the cannula and thrombophlebitis. These will be minimised by careful technique according to a standard SOP, cannula site inspection and replacement or removal where necessary. No more than 1ml/kg of blood will be drawn for research at any one time. All patients will be closely monitored so that clinical deteriorations can be identified at the earliest opportunity and appropriate therapy initiated. In general, the trial sites in Africa have considerable experience with this population and this will serve to minimise the risks to the patients and the trial.

A number of children will present as emergencies, where delay in study enrolment, and thus treatment, will not be practical or indeed ethical. We have received ethical approval to use a modified form of deferred consent; also recently used in the FEAST trial for which the deferred consent process was developed. It proposes to use a ‘two-stage’ consent process in this circumstance ^12, 13^. Verbal assent will be sought from parents or guardians by the admitting medical team, if it is considered that the full consent process would significantly delay treatment allocation, and consequently could be detrimental to the child’s health. Full consent will be sought once the child’s clinical condition has been stabilized. Caregivers will be provided with a brief verbal description of the trial and will be given the opportunity to “opt out” of clinical research. The clinician will later sign the verbal assent form, which will be filed with the consent form. If consent is withdrawn later no data from the subject will be used. Social science study of the consent processes used in FEAST found this to be acceptable to parents and health-care workers. As in the FEAST trial, if following an assent process a child died prior to full written consent, full consent would not be sought. This process of emergency consent was approved by multiple ethics research committees for FEAST and has been subsequently approved for use in a transfusion trial in Uganda and Blantyre ^14^.

## Discussion

At present, there is no clear evidence on what rate of rehydration is safe and of greatest benefit to a child admitted with severe dehydration due to gastroenteritis. Across Africa guidelines differ and many clinicians do not follow these since they are not evidence-based. In order to address this the data from this trial, generated in light of the results from the FEAST trial, are urgently needed. In addition, the GASTRO study will generate new data on whether bedside assessment of dehydration and its correction are reliable; on the safety of the current protocol recommended by WHO – since this data has never been provided by any previous study. The study will also provide more information about whether a slower rate of rehydration is safe and its efficacy on correction of dehydration **.**The planned research will be an important step in developing a future Phase III clinical trial. Results will inform the design of this trial. It will document fluid related adverse events in each of the groups, particularly cardiovascular, respiratory and neurological. It will also gather a series of clinical, biochemical and physiological data with the aim of generating robust definitions for a larger phase III trial (in particular for non-mortality endpoints).

### Implementation of the trial protocol

There were three main challenges faced when operationalizing this trial. The first was related to staff training and confidence in ensuring and documenting accurate fluid balance. Administering fluid in low resource settings most commonly involves hanging a 500ml bag of fluid and allowing it to run through a giving set, often until the bag is finished. Fluid volume and rate is difficult to control, particularly without the assistance of burettes and/ or infusion pumps, and staffing levels limit the ability to monitor and record fluid volumes infused and fluid output i.e. urine volumes, stool volumes and purging rates. Prior to initiating the study in Uganda, it was clear that WHO ‘Plan C’ is not being strictly adhered to. Most children were receiving 100ml/Kg but not following the split rates i.e. Step 1: 30mls/Kg over 30mins (or 1 hour if <1yr) followed by and Step 2: 70mls/Kg over 2.5 hours (or 5 hours if <1year). Instead, children were receiving 100ml/Kg at a non-specified rate with limited clinical reviews and observation. In order to address this challenge, we conducted a series of training activities, provided burettes, fluid balance charts and specific case report forms for bedside monitoring. Training for the paediatric departments and for the dedicated study teams was delivered on multiple occasions and retraining conducted if there were any questions or points of confusion. We revisited the case report forms to simplify these and minimise room for error.

Secondly, and predictably, the geographical and political context of this trial has limited recruitment rates to this study. A three-month (December 2016 to February 2017) national medical and nursing strike left Kenya without any public health care facilities and destroyed the public faith in their health system. As a result, numbers presenting to hospitals fell significantly, despite the fact that a small high dependency ward remained open to admissions. A second nursing strike was initiated in June 2017 and is ongoing. To increase the recruitment rate, the study was extended to an additional two sites in Uganda (Mbale and Soroti) and the sample size increased from 80 to 120 children in order to maintain balance across the sites and to protect the number of children having physiological assessments (ECHO and cytocam).

Finally, gastroenteritis is very seasonal and related to rains, and in 2017 Kenya experienced its worst drought for a decade and therefore admissions with gastroenteritis fell, again limiting numbers presenting and therefore restricting recruitment into GASTRO. Prospective research conducted as Kilifi County Hospital demonstrated that 20% of children hospitalised with AGE and severe dehydration (10% or more loss of body weight) temporarily fulfil anthropometric criteria for SAM (MUAC <11.5cm or WHZ <-3SD), but following rehydration they are reclassified as undernourished ^15^. Thus, the current recommendations have much wider implications with potentially 20% of children with severe dehydration secondary to AGE and without SAM receiving low volume low sodium rehydration, which may explain the poor outcomes that have been observed in the large case-control study Global Enteric Multicentre study (GEMS) ^2^.

### Trial status

Enrolment to the trial started in Kilifi, Kenya in November 2016 and in Uganda in January 2017 and is currently ongoing. There have been episodes of slow recruitment as a result of national strikes in Kenya, and a severe drought across East Africa resulting in higher numbers of children with under-nutrition fulfilling severe malnutrition anthropometric criteria and with 10% dehydration ^15^, as well as fewer children arriving at hospital with gastroenteritis. By 3 ^rd^ August 2017 56 children had been enrolled across all three centres.

### Protocol version changes

Version 1.0 was the original protocol submitted for ethical approval to SERU and was approved on 16 ^th^ August 2016.

Version 2.0 detailed minor grammatical changes requested by ICREC: an additional sentence in the consent form explaining the difference between the two arms and clarification of sample storage procedures.

Version 3.0 details additional blood sampling on day 7 and analysis of blood for cardiac enzymes and urine for electrolytes and osmolality.

The current version, Version 4.0 includes an additional PI (PO-O), an increase in sample size to 120, and the addition of two sites in Uganda (Mbale and Soroti).

## Abbreviations

AGE: Acute gastroenteritis; AVPU: Alert, voice, pain, unresponsive (system of recording patient’s level of consciousness); BIA: Bioimpedance analysis; CRF: Case report form; ETAT: Emergency triage assessment and treatment; FEAST: Fluid Expansion As a Supportive Therapy; GEMS: Global Enteric Multicentre study; GI: Gastrointestinal; ICREC: Imperial college research ethics committee; IPR: Industrial property act; KWTRP: KEMRI Wellcome Trust Project; MRC:Medical research council; MRHIRC: Mbale Regional Hospital Institutional Review Committee; MUAC: Mid-upper arm circumference; NGT: Nasogastric tube; ORS: Oral rehydration solution; SERU: KEMRI Scientific ethics review unit; US: Ultrasound; WHO: World Health Organization.

## Supplementary material

Supplementary File 1: Patient information sheet and consent form.

Supplementary File 2. Data and Safety Monitoring Board Charter.

Supplementary File 3. GASTRO Statistical Analysis Plan.
