# Supplementary material for: Gastroenteritis Aggressive Versus Slow Treatment For Rehydration (GASTRO). A pilot rehydration study for severe dehydration: WHO plan C versus slower rehydration
Source: Wellcome Open Res. 2017 Aug 10;2:62. [Version 1] doi: 10.12688/wellcomeopenres.12261.1 (PMC5571888; doi:10.12688/wellcomeopenres.12261.1)
Supplement: Supplementary file 3 [file wellcomeopenres-2-13274-s0002.tgz › f7e84a1d-033f-495c-830d-3f6c4efb5df5.pdf]

Statistical Analytical Plan	**Version:** 1.0<br>**Supercedes:** None<br>**Effective Date:**

GASTROENTERITIS AGGRESSIVE VERSUS SLOW TREATMENT FOR REHYDRATION (GASTRO) STUDY

	NAME	SIGNATURE	DATE
**Preparer**	**Kirsty Houston**		
**Reviewing Authority**	**Kath Maitland**		
**Study statistician**	**Elizabeth George**		

1. PROTOCOL DETAILS

Protocol Title: A Pilot rehydration study for severe dehydration: WHO plan C versus slower rehydration?

Study short title: GASTRO – Gastroenteritis Aggressive versus Slow Treatment for Rehydration

SERU/SSC Protocol number: KEMRI/SERU/CGMRC/053/3299

Principal Investigators and Contacts

1. Kirsty Houston (coordinator) – kirstyhouston85@gmail.com, 0702780591
2. Kath Maitland

2. INTRODUCTION

The current WHO rehydration management guidelines (Plan C) for children with gastroenteritis and severe dehydration are widely practiced in resource-poor settings, despite never having been tested in a clinical trial. A recent audit of outcome of acute gastroenteritis (AGE) at Kilifi County Hospital, Kenya noted that 10% children with gastroenteritis required high dependency care managed on WHO Plan C protocol (mortality up to 20%) and a number developed fluid-related complications such as signs of cardiovascular collapse or neurological compromise. The fluid resuscitation trial, FEAST, conducted in children with severe febrile illness raised concerns regarding the safety of rapid intravenous rehydration therapy in other illnesses such as acute gastroenteritis and warrants more detailed examination for assessment and quantification of myocardial performance indices and haemodynamic response to therapy using non-invasive echocardiography imaging. We therefore proposed a pilot Phase II study to evaluate initial fluid management in children admitted to hospital with severe dehydration secondary to gastroenteritis. We will compare standard aggressive rehydration (WHO plan 'C' – 100mls/kg over 3 hours, plus boluses for those with shock) versus a slower rehydration regimen (given over 8 hours). Formal sample sizes have not been calculated for this in-depth pilot study. We aim to study 120 children in total (60 in each arm), which is realistic to obtain an idea about major adverse effects. It is also realistic given the timeframe and funding available for the study. The focus of this pilot is firstly to document adverse events, particularly related to cardiovascular compromise and neurological sequelae and secondly, to gather a series of useful clinical, biochemical, and physiological data on initial assessment of severity of dehydration, and response to treatment of children by intravenous (IV) rehydration. Results from this pilot will contribute to generating robust definitions of outcomes for a larger phase III trial (in particular for non-mortality endpoints).

3. STUDY OBJECTIVES

a) General Objectives:

To compare the current standard WHO plan 'C' rehydration protocol with a strategy that aims to give a slower rehydration regimen using the same total volume (100ml/kg of Ringers Lactate) over 8 hours, irrespective of age

b) Specific Objectives:

i. Document adverse events, particularly related to cardiovascular compromise and neurological sequelae.
ii. Gather a series of clinical, biochemical, and physiological data on:
    1. Initial assessment of dehydration
    2. Response to treatment of children by IV rehydration.
iii. To inform robust definitions of outcomes for a larger phase III trial

4. DESIGN

a. Overview

Multi-centre, randomised, open label controlled pilot study to demonstrate safety and efficacy of a slower rehydration strategy.

b. <u>Expected Sample Size</u>

We have not calculated formal sample sizes. We aim to study 120 children (60 in each arm); this will enable a sample size to provide sufficient pilot data (clinical and physiological) on the main outcomes given the timeframe of the study and potential limiting any exposure to a potentially inferior treatment strategy should this become evident during the conduct of the study. Within this, we aim to recruit a minimum of 40 patients in Kilifi, Kenya.

i. <u>Inclusion Criteria</u>

- Children, aged 60 days to 12 years
- Acute gastroenteritis (> 3 loose stools/day)
- Signs of severe dehydration (as per WHO definition – unable to drink or AVPU <A, with sunken eyes and reduced skin pinch (<2seconds) and an inability to take or retain oral fluids), with or without shock.
- Shock will be defined as; a patient with all of the following: cold peripheries with a weak and fast pulse (rate not specified) and a capillary refill time >3 seconds.

ii. <u>Exclusion Criteria</u>

- Severe malnutrition (kwashiorkor or MUAC <11.5cm)
- Diarrhoea lasting more than 14-days
- Known congenital heart disease

5. <u>STUDY SCHEMA</u>

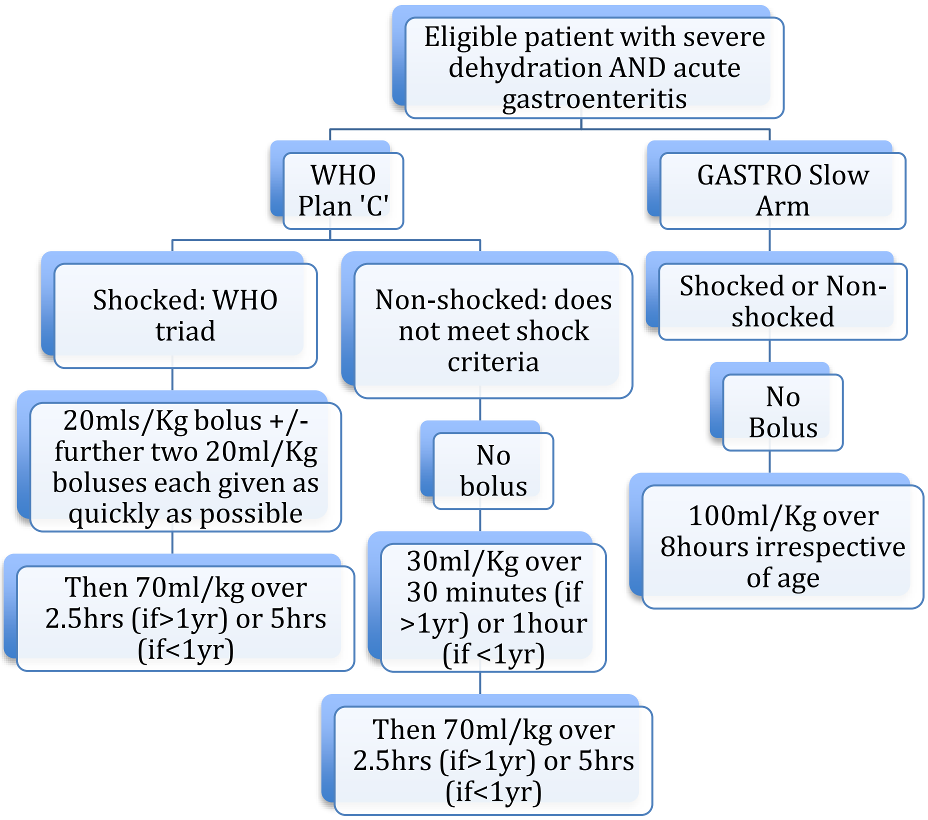

6. OUTCOME MEASURES

a. Primary (safety) outcome measure:

i. Frequency of pre defined, fluid related, significant adverse events including; mortality, cardiovascular collapse, raised intracranial pressure, pulmonary oedema and allergic reaction.

b. Secondary (efficacy) endpoints

i. Correction of dehydration
ii. Time to pass urine
iii. Dysnatraemia at 8 hours
iv. Time to tolerate oral fluids/ feeds
v. Time to discharge
vi. Readmission rate

c. Physiological Endpoints (Kilifi only)

i. ECHO
ii. Bioelectrical impedance
    1. Change in Phase Angle and prediction marker
    2. Correlation with clinical hydration
iii. CytoCam
    1. POEM Score over time

7. STATISTICAL METHODS

a. Pooling of Data for Analyses

The clinical study will be conducted under a common protocol for each investigational site with the intention of pooling the data for analysis. Every effort will be made to promote consistency in study execution at each investigational site. The analysis of homogeneity will be conducted under the assumption that each site will have enrolled a minimum of 20 ITT subjects, 10 in each arm.

b. Statistical methods

i. **Significance testing –** all statistical tests will use a significance level of 0.05. Two-tailed tests will be performed for all analyses that use statistical testing. Confidence intervals will be presented with 95% degree of confidence.
All p-values will be rounded to 3 decimal places. All p-values that round to 0.000 will be presented as '<0.001' and p-values that round to 1.000 will be presented as '>0.999'. Any p-value ≤0.05 will be considered statistically significant.
Data summarization – Summary statistics will consist of the number and percentage of responses in each category for discrete variables, and the mean, median, standard deviation (SD), and interquartile range (IQR) for continuous variables.
All mean and median values will be formatted to one more decimal place than the measured value e.g. measured values 3.4, 3.5, mean 3.45

Standard deviation values will be formatted to two more decimal places than the measured value e.g. measured value 2.5, standard deviation 1.34
Minimum and Maximum values will be presented to the same number of decimal places as the measured value.
All percentages will be rounded to one decimal place (the number and percentage will be presented).
All analysis and summary tables will have the population sample size for each treatment group in the column heading.

ii. **Missing Data** – The investigators were instructed to obtain complete CRFs. Missing data will not be imputed.

iii. **Software – Stata**

c. Populations – the intention to treat (ITT) population includes all patients who were assigned a randomisation number. The ITT population is considered the main analysis population. Per protocol will include all children who received fluids as per randomization arm.

d. Statistical Analyses

i. Demographic and Baseline Characteristics

These will be presented by arm:

1. **Enrolment and eligibility**
   a. Intake by site
   b. Number of children that assented instead of consented
   c. Details of children excluded from subsequent analyses
2. **Adherence to the allocated fluid management strategy**
   a. Number of children given WHO Plan C vs. Slow arm
   b. Number of shocked children in each arm (and number receiving a bolus)
   c. Fluid received in each arm and by age at the following time points: 1hr/ 2hr/ 4hr/ 6hr/ 8hr/ 24hr, presented as mls/kg/hr
   d. Number of protocol deviations in this study
3. **Demographics**
   a. Sex (%): male/ female
   b. Age: mean (SD), median (IQR), range, number (%) in categories (>2months to ≤12months, >12months to ≤24months, >24mnths to ≤36months, >36months)
   c. Weight: mean (SD), median (IQR), number (%) in categories (<9Kg, 9-12Kg, >12Kg)

d. Mid Upper arm Circumference (MUAC): mean (SD), median (IQR), range, number (%) in categories: 11.5-13, 13.1-15, >15)

4. **Triage at admission**
    a. Axillary temperature: mean (SD), median(IQR), range, number (%) in categories (<36, 36 – 38.9, >39 degrees Celsius)
    b. Respiratory Rate: mean(SD), median(IQR), range, number (%) with 'fast breathing'/tachypneoa (<1yr: >50 breaths/min; 1-4yrs: >40 breaths/min; >5 yrs: >60 breaths/min)
    c. Oxygen Saturation: mean (SD), median (IQR), range, number (%) with hypoxia (<90 % Hypoxaemia, 90 – 95%, >95%)
    d. Heart rate: mean (SD), median(IQR), range, number (%) with <80bpm 'bradycardia', number (%) with 'severe tachycardia' (<1yr: >180bpm; 1 – 5yrs: 160bpm; >5yrs: >140bpm).
    e. Capillary refill time: median (IQR), number (%) in categories (<3 and ≥4 seconds)
    f. Temperature gradient : number (%) with temperature gradient
    g. Weak pulse: number (%) with weak radial pulse
    h. Conscious level : number (%) in categories (alert, prostate, coma)
    i. Fits/convulsions: number (%) with fits and/or convulsions.
    j. Systolic Blood Pressure: mean(SD), median(IQR), range, number (%) with Severe hypotension (defined as <12 months= <50mmHg; 12 mths to 5 yrs = <60mmHg, >5 yrs = <70mmHg) and Moderate Hypotension (defined as: <12 months= 50-75mmHg; 12 mths to 5yrs = 60-75mmHg; > 5 yrs = 70- 85mmHg)
    k. Respiratory distress: number (%) with respiratory distress.

5. **Lab results at admission**
    a. Lactate: mean(SD), median(IQR), range, number (%) in categories (≤ 5, >5 mmol/L)
    b. Glucose: mean (SD), median(IQR), range, number (%) in categories (<2.5 [hypoglycaemia], 2.6 – 10, >10 mmol/L [hyperglycaemia])
    c. Haemoglobin: mean (SD), median(IQR), range, number (%) in categories (<6, 6-11, >11g/mL)
    d. HIV status: number (%) in categories (positive, negative, unknown)
    e. Malaria – either slide or rapid diagnostic test positive
    f. Biochemistry/ blood gas:
        a. Sodium: Hyponatraemia <125mmol/l

b. Potassium: Hyperkalaemia >5.5mmol/l; Hypokalaemia <3mmol/l
c. Chloride (Cl) (mmmo/L)
d. pH: Severe Acidaemia <7.2 (Kilifi only)
e. pCO2: Hypercapnia >6 mmHg (Kilifi only)
f. Haematocrit (Hct) (%)
g. Bicarbonate HCO3 (mmol/L) (Kilifi only)
h. Base Excess: Acidosis: <-8 mmol/L (Kilifi only)
i. Anion Gap (mmo/L) (Kilifi only)

6. **Description of follow-up**
   a. Number of children whose vital status at 48hrs is known
   b. Number of children for whom consent was withdrawn before 48 hours
   c. Number of children who absconded before 48hrs
   d. Number of children lost to follow up (LTFU) by Day 7 review. And, where children are LTFU, number of children whose vital status is known.
   e. Of the children who completed Day 7 review, how many completed it i) on day 7, ii) after day 7.

ii. Primary Safety Analyses

1. The proportion of children with a pre-specified significant adverse event at 48hours in the Plan C and the GASTRO slow arm will be compared using an unadjusted chi-squared test on 1 degree of freedom Risk difference (95% CI) and risk ratio (95% CI) will also be presented.

iii. Secondary (Efficacy) Analyses

1. Correction of dehydration – time to correction of dehydration will be compared between the two groups using Kaplan Meier and log rank tests.
2. Time to pass urine will be compared between two groups using Kaplan Meier and log rank tests.
3. Dysnatraemia (defined as <135 and >143) at 8 hours will be compared between the two groups using the Chi-squared test.
4. Time to tolerate oral fluids/ feeds will be compared between two groups using Kaplan Meier and log rank tests.
5. Readmission rate by Day 7 will be compared between the two groups using the Chi-squared test.

iv. Physiological study Analyses (Kilifi and Mbale data only)

1. **Bioelectrical Impedance –** Phase angle and prediction markers will be compared between the two groups using the students t test. These will be correlated with clinical hydration status.
2. **CytoCam** – POEM score will be compared between the two groups using the Chi-squared test.
3. **ECHOcardiography –** will be analysed using specific software in Brisbane, Australia. Specific data analysis to be specified in separate document.

4. **Cardiac Biomarkers**

v. Subgroup analyses

1. All analyses will be repeated in strata according to: age, nutritional status (mild/ moderate/ no malnutrition); severity clinical: (number of feature of impaired perfusion; numbers of features of dehydration), conscious level, respiratory distress, and laboratory feature (**cardiac biomarkers of 'strain' at admssion, dysnatraemia, acute kidney injury, severe anaemia, bacteraemia)** and HIV status.
