# Supplementary material for: Gastroenteritis Aggressive Versus Slow Treatment For Rehydration (GASTRO). A pilot rehydration study for severe dehydration: WHO plan C versus slower rehydration
Source: Wellcome Open Res. 2017 Aug 10;2:62. [Version 1] doi: 10.12688/wellcomeopenres.12261.1 (PMC5571888; doi:10.12688/wellcomeopenres.12261.1)
Supplement: Supplementary file 1 [file wellcomeopenres-2-13274-s0000.tgz › f35e9fa3-078f-4b87-a2c2-883bc24a6ed2.doc]

**KEMRI Wellcome Trust Research Programme: Patient Information and Consent Form**

**Gastroenteritis Aggressive versus Slow Treatment for RehydratiOn: a pilot study (GASTRO)**

A Pilot rehydration study for severe dehydration: WHO plan C versus slower rehydration

| **Institution** | **Individuals** |
| --- | --- |
| **KEMRI** | Nchafatso Obonyo, Ayub Mpoya |
| **KEMRI & Imperial College, UK** | Kathryn Maitland |
| **Imperial College** | Kirsty Houston, Jack Gibb |
| **Mbale Regional Referral Centre** | Dr Peter Olupot-Olupot |

**Introduction**

Your child has been examined by the doctor and found to have signs of severe fluid loss (called dehydration) as a result of an illness which causes diarrhoea and vomiting called gastroenteritis. We need to admit your child to hospital, take some blood tests and treat them by starting a drip (show) to replace these losses. This is part of the normal care for a child with severe dehydration. Once they are able to drink and keep down feeds we can then stop the drip treatment.

**What is this research about?**

In this research we aim to find out whether we need to replace the fluid losses rapidly or more slowly That means that the drip will run either quickly over 3-6 hours or more slowly over 8 hours. The fluid treatment (salt and water) in this research is what all children with dehydration usually recieve with good effect in other parts of the world. We want to find out if slower fluid administration is better for children in Africa. Since this is not known at the moment, we would like to learn this through allocating up to 120 children aged 2 months to 12 years with dehydration as a result of diarrhea and vomiting into one of two treatment groups:

1) Receiving nationally recommended treatment, with additional fast fluid in the first hour if your child is very sick
2) Receiving recommended treatment but more slowly, over 8 hours, and with any additional fluid required early on (if your child is very sick) being included in the total volume

Which of the two types of treatment a child is given will be decided by chance.

**What is KEMRI?**

KEMRI is a government organisation that carries out medical research. Research is different from normal treatment because research aims to find better ways of preventing and treating illness in the future for everybody’s benefit. We are asking permission for your child to participate in this research study.

**What will it involve for me/my child?**

1. For the children in the group receiving fluid quickly which is currently recommended by national guidelines, this will run over 3-6hours depending on their age. They may also receive an extra one or two ‘rapid boluses’ or large doses of fluid in the first hour.
2. For the children in the slow arm, they will receive the same volume of fluid by drip but given more slowly over 8 hours, and no initial ‘rapid boluses’ or large doses of fluid in the first hour.
3. All children will be closely watched to decide whether to make any changes to treatment or to prescribe more fluid. This monitoring will be through regular checks by the nurses. Currently, we are taking 1 teaspoon (5mls) of blood at admission and half a teaspoon (2.5ml) the day after admission as part of routine clinical care. In this study, we will take an additional teaspoon of blood on admission and a total of approximately a teaspoon (4.5mls) at 8 and 24 hours after admission.
4. As part of normal care the nursing staff place a soft tube called a catheter into the bladder in order to help us find out whether the fluids we are giving are having a good effect on urine output. From this we will take urine samples at 1hour, 8 hours and 24hours after admission. This will involve no additional discomfort to your child.
5. For some children, we will also look at other ways of how your child is responding to the fluids by using a small machine called an echo which is placed on the chest for 3-5 minutes to see how blood is pumped around the body. This will happen 5 times during the study (in hospital). We have used these before in lots of children – they wont hurt your child or will not involve any risk to your child.
6. For some children, another machine (BIA) will be attached to your childs arm and foot for 1 minute to see how well the fluid loss is being replaced. This machine will not hurt your child or involve any risk to them. This will be done 3 times during the study (while in hospital) and once more at the follow up appointment.
7. For some children, another machine (CytcoCam) which is a specialised camera, will be placed inside your childs mouth, on their cheek, to take pictures of the blood flowing around your childs body. This will be done four times during the study (3 times in hospital and once more at follow up). This does not involve any risk to your child and will not hurt them.
8. We will also ask you to come back for follow up to check on your child’s progress 7 days post admission, in the hospital clinic. This will also include measuring your childs weight and, for some children, taking another measurement with the machines again. At this follow up appointment we will take one further blood sample (one teaspoon of blood) and one more urine sample (this time the urine sample will involve passing urine into a container and will not involve insertion of a catheter).

**Are there any risks or disadvantages to me/my child of taking part?**

- Our priority for every participant is their well-being.
- Giving fluids by drips is used extensively used in children in Kenya and other parts of the world. We will be monitoring your child very closely. If for any reason the doctor thinks that it is not in your child’s best interest to be in the trial, then s/he will not be enrolled in the trial but will be given normal standard of care. There are no costs for being included in the trial.
- Taking blood from the arm causes a small amount of pain, *bruising, swelling, discomfort and minimal chance of infection. If this happens, we will provide treatment.* The amount taken is too small to affect your child’s health.
- Inserting a catheter involves a small amount of pain during the procedure, and a minimal chance of infection. If this happens we will provide treatment. Once the catheter is in place your child should not have any discomfort. The catheter will allow us to measure your childs urine output much more accurately.
- You will be asked to bring your child back for follow up at this clinic, but we will pay for the costs of your transport for this visit. One follow up visit will take up approximately 30minutes of your time.
- An independent committee will monitor this research continuously to ensure participants safety and rights are respected at all times.

**Are there any advantages to me/my child of taking part?**

Your child will get close observation and our usual standard treatments during the trial, and by taking part your child may help us improve the care of children who have serious dehydration in the future. We will provide appropriate treatment for any complications that occur during the time your child is in the study. However, for conditions that we are unable to manage, identified at time of admission, or new illnesses that occur after child is enrolled into the study, we will ensure that we refer these to the appropriate government health facilities.

We will pay for your transport from home to bring your child back for follow up at 7 days. We will pay for your transport from hospital to your home and back to the clinic so you can attend this visit (between Ksh. 100 and Ksh.500 depending on where you come from and the fare amount you spend). We will also compensate you for any wages lost for the time you attend this clinic visit at the rate of Ksh.350 per day of follow-up visit. After this time your child, if there are any on-going medical problems will be referred to/treated by the government services.

**What happens if I refuse to participate?**

All participation in research is voluntary. You are free to decide if you want your child to take part. Your child will still receive the recommended standard of care if they do not take part. If you do agree you can change your mind at any time and withdraw your child from the research. This will not affect your child’s care now or in the future.

**What happens to the samples?**

Blood and urine samples will be processed in labs here in Kilifi. Samples will be stored indefinitely in the KEMRI CGMRC repository. Individual names will be removed and will be replaced by codes, so that information cannot be linked to participants. Any future research done on these samples must first be approved by a national independent expert committee, to ensure that participants’ safety and rights are respected.

**Who will have access to information about me/my child in this research?**

All our research records are stored securely in locked cabinets and password protected computers. Only a few people who are closely concerned with the research will be able to view information from participants.

In future, information collected or generated during this study may be used to support new research by other researchers in other countries in Africa and internationally on diarrhoea. In all cases, we will only share information with other researchers in ways that do not reveal individual participants’ identities. For example, we will remove information that could identify people, such as their names and where they live, and replace this information with number codes. Any future research using information from this study must first be approved by a local or national expert committee to make sure that the interests of participants and their communities are protected.

**Who has allowed this research to take place?**

All research conducted in *Kenya* is approved by national independent expert committees to make sure the research is conducted properly and that trial participants’ safety and rights are respected.

**What if I have any questions?**

You may ask any of our study staff questions at any time. You can also contact those who are responsible for the care of your child in this research.

**Dr Kathryn Maitland**

**KEMRI- Wellcome Trust**

**P.O.Box 230-80108 Kilifi. Telephone: 0715461761**

If you want to ask someone independent anything about this research please contact:

**Community Liaison Manager, KEMRI – Wellcome Trust P.O.Box 230-80108 Kilifi.**

**Telephone: 0723342780 or 041 7522 063**

***Or***

**The Secretary – KEMRI Scientific and Ethics Review Unit (SERU)**

**P. O. BOX 54840-00200 Nairobi**

**Tel number: 020 272 2541 Mobile: 0722205901 or 0733400003 check number**

**This research is supported by the Imperial College, London who will pay for any trial related treatment or compensation in the unlikely event of any injury resulting from this study**

**Gastroenteritis Aggressive versus Slow Treatment for RehydatiOn: a pilot trial (GASTRO)**

**To be completed after the patient has read or been read to the patient information sheet**

I, [being a parent/guardian of ________________________________ (name of child),] have had the research explained to me. I have understood all that has been read/explained and had my questions answered satisfactorily.

**Please insert the boxes below or add others where relevant**

** Yes**	*please tick*	**I agree to participate/allow my child to take part in this research**

** Yes**	*please tick*	**I agree to samples being stored and used for future research**

I understand that I can change my mind at any stage and it will not affect to me/my child in any way.

**Subject/Parent/guardian’s signature**: ____________________________**Date** ___________

**Subject/Parent/guardian’s name:** _____________________________ **Time** ____________

(Please print name)

------------------------------------------------------------------------------------------------------------------------------------------

***Where parent/guardian cannot read, ensure a witness****** ***observes consent process and signs below:***

I attest that the information concerning this research was accurately explained to and apparently understood by the subject/parent/guardian and that informed consent was freely given by the subject/parent/guardian.

**Witness’ signature:** _____________________________________ **Date** _____________

**Witness’ name**: _____________________________________ **Time** ______________

**A witness is a person who is independent from the trial or a member of staff who was not involved in gaining the consent.*

Thumbprint of the parent as named above if they cannot write: _________________________

I have followed the study SOP to obtain consent from the [participant/guardian]. S/he apparently understood the nature and the purpose of the study and consents to the participation [of the child] in the study. S/he has been given opportunity to ask questions which have been answered satisfactorily.

**Designee/investigator’s signature:** ____________________________ **Date** ____________

**Designee/investigator’s name :** _____________________________**Time** ____________

(Please print name)

**Note:** One signed original copy to be kept in GASTRO trial master file and the other signed copy to be given to parent/guardian.

Parental Assent Form for Emergencies/Critical Sick children

**Gastroenteritis Aggressive versus Slow Treatment for Rehydration: a pilot trial (GASTRO)**

**(Please see guidance below)**

| Child’s Initials |  |  |  |
| --- | --- | --- | --- |

| Kemri Serial Number |  |  |  |  |  |
| --- | --- | --- | --- | --- | --- |

*We recommend brief discussion with the parents/guardians should include the following phrases.*

- Your child is being examined by a doctor at the moment and all efforts being made to diagnose and stabilize the child. We need to admit your child to hospital.
- We are working for KEMRI. We work hand in hand with the MoH to make sure that each child is treated to the best of our ability.
- Your child is very ill and needs fluids by a drip (indicate). At the moment we need to know whether the current treatment recommendations to give this very fast or the one we are suggesting which give fluid more slowly will work better. We do this through research.
- An independent committee in Kenya that is run by the Government and a committee in Kilifi have looked carefully at this and agreed that the research is important, that it should be safe for you to participate and that the study will be conducted properly.
- We would like your child to participate in research for us to learn the best way to give fluids to very dehydrated children. We will explain in detail about this later we are now asking whether you are happy us to proceed with this research treatment for your child.
- Do you agree for your child to take part in this research? You can say no and your child will still receive the same level of care with the government recommended treatment.

| Parent/Guardian assents to research? | Yes / No |
| --- | --- |

| Doctor’s or nurse’s signature | Print name | Time | | | | Date | | | | | | | | |
| --- | --- | --- | --- | --- | --- | --- | --- | --- | --- | --- | --- | --- | --- | --- |
|  |  | *H* | *H* | *M* | *M* | *D* | *D* | *M* | *M* | *M* | **2** | **0** | *Y* | *Y* |

**Guidance**

Caregivers will be provided with a brief verbal description of the trial and given the opportunity to “opt out” of clinical research. If the parent/guardian agrees the clinician or nurse will sign the verbal assent form. **The parent/guardian or child (aged 8-12 years) will NOT be required to sign the emergency assent form.**

Once the participant’s condition has stabilized, full informed consent will be sought from the parent/guardian and parent asked to sign the informed consent form.
