# Supplementary material for: Gastroenteritis Aggressive Versus Slow Treatment For Rehydration (GASTRO). A pilot rehydration study for severe dehydration: WHO plan C versus slower rehydration
Source: Wellcome Open Res. 2017 Aug 10;2:62. [Version 1] doi: 10.12688/wellcomeopenres.12261.1 (PMC5571888; doi:10.12688/wellcomeopenres.12261.1)
Supplement: Supplementary file 2 [file wellcomeopenres-2-13274-s0001.tgz › 16ef57f9-dc05-4a7c-9dd3-5a5430809bf6.doc]

A Pilot rehydration study for severe dehydration: WHO plan C versus slower rehydration

**ISRCTN** 67518332

**ICREC** 16IC3388

**SERU** 053/3299

**Independent Data Safety Monitoring Board**

**Committee Charter**

**Version 1 12^th^ Oct-2016**

## IDMC CHARTER FOR [GASTRO TRIAL]

| Content  Guidance | | charter details |
| --- | --- | --- |
| 1. Introduction | |  |
| Name | | A randomised trial of intravenous rehydration strategies in African children with severe dehydration secondary to Gastroenteritis (GASTRO) |
| Objectives of trial, including interventions being investigated | | **The GASTRO trial addresses the question of whether s**lower rehydration is equally effective in rehydration but associated with fewer fluid related adverse effects  **General Objectives:**  To compare the current standard WHO plan ‘C’ rehydration protocol with a strategy that aims to give a slower rehydration regimen using the same total volume (100ml/kg of Ringers Lactate) over 8 hours, irrespective of age  Specific Objectives:   1. Document adverse events, particularly related to cardiovascular compromise and neurological sequelae. 2. Gather a series of clinical, biochemical, and physiological data on: a) Initial assessment of dehydration b) Response to treatment of children by IV rehydration. 3. To inform robust definitions of outcomes for a larger phase III trial   GASTRO is a two arm open randomised controlled comparison of two intravenous rehydration strategies: 1) WHO Plan C 100mls/Kg over 3hours (6hours if aged <1year), with boluses for children who meet WHO criteria for shock, versus 2) Conservative arm will receive 100mls/Kg over 8hours, regardless of age (no boluses). 120 children will be recruited and randomised 1:1 allocation. The trial will be conducted in Kilifi County Hospital, Kenya, and Mbale and Soroti Regional Referral Hospitals, Uganda. |
| Outline of scope of charter | | The purpose of this document is to describe the membership, roles, responsibilities and decision-making of the independent DSMB for the this trial |
| 2. Roles and responsibilities | |  |
| A broad statement of the aims of the committee | | To safeguard the interests of trial participants, monitor the main outcome measures including safety and efficacy, and monitor the overall conduct of the trial. |
| Terms of reference | | The DSMB should receive and review information on the progress and accruing data of this trial and provide advice on the conduct of the trial |
| Specific roles of IDMC | | Interim review of the trial’s progress including updated figures on recruitment, data quality, adherence to protocol treatment and follow-up, and main outcomes and safety data. Specifically, these roles include to:   - monitor evidence for treatment differences in the main efficacy outcome measures - monitor evidence for treatment harm (e.g. toxicity, SAEs and SARs, deaths) - assess the impact and relevance of external evidence if any - decide whether to recommend that the trial continues to recruit participants or whether recruitment should be terminated for all or some participant subgroups - assess data quality, including completeness (and by so doing encourage collection of high quality data) - maintain confidentiality of all trial information that is not in the public domain - monitor recruitment figures and losses to follow-up - monitor compliance with the protocol by participants and investigators |
| 3. Meetings | |  |
| Timing of the IDMC meeting | | The IDMC will first meet after 40 patients have enrolled in the trial, to discuss the protocol, the trial, the plan for interim analysis, future meetings, and to have the opportunity to clarify any aspects with the Chief Investigator (CI). |
| IDMC contract | | IDMC members will not formally sign a contract but should formally register their assent to join the group by confirming  (1) that they agree to be on the DSMB and  (2) that they agree with the contents of this Charter.  Any competing interests should be declared at the same time. |
| 4. Composition | |  |
| Membership of the IDMC | | The members of the IDMC for this trial are:  1/ **Professor Tim Peto** (Chairman)  Nuffield Dept of Medicine, John Radcliffe Hospital  Headington, Oxford OX3 9DU  2/ **Professor Grace Irimu** (Paediatrician/Clinical Research)  KEMRI Wellcome Trust Research Programme, Nairobi  Clinician scientist, previous clinical trial conduct  3/ **Professor Elizabeth Molyneux** (Clinician)  Professor of Paediatrics; Malawi-Liverpool-Wellcome Trust; Clinical Research Programme; Blantyre, Malawi  Guidelines for severely ill children and extensive Clinical trials experience: chair of numerous trial steering groups and previous membership of DSMB.  **4/ Ms Elizabeth George (DSMB Statistician)**  Statistician MRC Clinical Trials Unit at UCL, 125 Aviation House, Kingsway, London, WC2B 6NH |
| IDMC Chairman: choice and role. | | The Chair has been nominated and agreed by the trial team at KCTF and Imperial College (sponsor).  Professor Timothy Peto (Chairman) has previous experience of Chairing meetings of DMCs, and will be able to facilitate and summarise discussions. He was chairman of the FEAST trial and committee recommending early termination of trial recruitment. |
| The responsibilities of the Kilifi Clinical Trials Facility (KCTF) | | An administrator from KCTF will prepare and circulate the agenda of the meetings and to take the minutes. |
| The responsibilities of the Chief Investigator and other members. | | The trial management team will help the trial statistician to produce the report to the DSMB. This will be available to the IDMC at least one week ahead of the meeting.  The Chief Investigator and other members of the trial team will attend the open meeting of the DSMB and will be responsible for drafting a response to the DSMB. |
| 5. Relationships | |  |
| Relationships with Chief Investigators and other trial committees, sponsors and regulatory bodies. | | The responsibilities of each trial group are detailed in the protocol.  If required, the reports from the DSMB will be sent to the ethics committees or other regulatory authorities. |
| Payments to IDMC members | | No other payments or rewards are given. |
| Need for IDMC members to disclose information about any competing interests | | Competing interests should be disclosed. |
| 6. Organisation of meetings | |  |
| Organisation of the IDMC meetings | | A mixture of open and closed sessions will be held.  Closed sessions will only be attended by IDMC members and the unblinded trial statistician.  In open sessions, all those attending the closed session may be joined by the CI(s), other members of the trial team  What decisions will be made during the open session?  1. An administrative session for members only.  2. Open session: Introduction and any “open” parts of the report. Unblinded results will not be discussed in this session.  3. Closed session: IDMC discussion of “closed” parts of the report and, if necessary, the trial statistician will attend part of these discussions |
| 7. Trial documentation and procedures to ensure confidentiality and proper communication | | |
| Content of material to be available in open sessions | Accumulating information relating to recruitment and data quality (e.g. data return rates, protocol and treatment compliance) will be presented.  Total numbers of events for the primary outcome measure and other secondary outcome measures will be presented (but not be summarised by treatment group)  SAE’s and/or toxicity details based on pooled data will be presented and total numbers of events for the primary outcome measure and other outcome measures may be presented, at the discretion of the IDMC. | |
| Content of material to be available in closed sessions | In addition to all the material available in the open session, the closed session material will include efficacy and safety data by treatment group. | |
| If DSMB will be blinded to the treatment allocation | The DSMB will not be blinded to treatment allocation. | |
| The people who will see the accumulating data and interim analysis | The accumulating data and interim analysis by randomised group will be seen by the DSMB members and trial statistician(s).  IDMC members do **not** have the right to share confidential information with anyone outside the IDMC, including the CI. | |
| To whom the DSMB will communicate the decisions/ recommendations that are reached | The DSMB reports its recommendations in writing to the trial PI and the trial statistician. | |
| Whether reports to the DSMB be available before the meeting or only at/during the meeting | The DSMB should receive the report at least 1 week before any meetings. | |
| 10. After the trial |  | |
| Publication of results | The Trial Management Group will form the basis of the Writing Committee, and will advise on the nature of publications. Members of the TMG will be listed, and contributors will be cited by name, provided that this does not conflict with journal policy  At the end of the trial there may be a meeting (by teleconference, if necessary) to allow the DSMB to discuss the final data with the writing committee to give advice about data interpretation.  The main trial results will be published in a correct and timely manner. | |
| The information about the IDMC that will be included in published trial reports | DSMB members will be named and their affiliations listed in the main report, unless they explicitly request otherwise. A brief summary of the timings and conclusions of IDMC meetings should be included in the body of this paper. | |
| Whether the IDMC will have the opportunity to approve publications, especially with respect to reporting of any DSMB recommendation regarding termination of a trial | The DSMB will be given the opportunity to read and comment on publications before submission. The commenting period will usually be 2 weeks. | |

## Annexe 1: Agreement and potential competing interests form

**GASTRO trial (ISRCTN** 67518332**):** Agreement to join the Independent Data Monitoring Committee and disclosure of potential competing interests

Please complete the following document and return to the GASTRO Trial Manager.

(please initial box to agree)

|  | I have read and understood the IDMC Charter version 1, dated _12_/_10_/_2016_ |
| --- | --- |
|  | I agree to join the Independent Data Monitoring Committee for this trial |
|  | I agree to treat all sensitive trial data and discussions confidentially |

The avoidance of any perception that members of a IDMC may be biased in some fashion is important for the credibility of the decisions made by the IDMC and for the integrity of the trial.

Possible competing interest should be disclosed via the KWTRP CTF. In many cases simple disclosure up front should be sufficient. Otherwise, the (potential) IDMC member should remove the conflict or stop participating in the IDMC. **Table 1** lists potential competing interests.

|  | **No,** I have no competing interests to declare |
| --- | --- |
|  | **Yes,** I have competing interests to declare (please detail below) |

| Please provide details of any competing interests: | |
| --- | --- |
|  |  |
|  |  |
|  |  |

Name: ___________________________

Signed: __________________________	Date: ______________

##### Table 1: Potential competing interests

- Stock ownership in any commercial companies involved

- Stock transaction in any commercial company involved (if previously holding stock)

- Consulting arrangements with the Sponsor (including CI for other MRC trials)

- Frequent speaking engagements on behalf of the intervention

- Career tied up in a product or technique assessed by trial

- Hands-on participation in the trial

- Involvement in the running of the trial

- Emotional involvement in the trial

- Intellectual conflict e.g. strong prior belief in the trial’s experimental arm

- Involvement in regulatory issues relevant to the trial procedures

- Investment (financial or intellectual) or career tied up in competing products

- Involvement in the publication in the form of authorship

**Note:** This IDMC template was developed using MRC CTU template IDMC Charter v2.01, 13-Mar-2006; from DAMOCLES IDMC Charter template v1, Feb 2005

## Annexe 2: Agreement and confidentiality agreement for observers

# Independent Data Monitoring Committee for GASTRO trial: Agreement to attend the Data Monitoring Committee and treat all information confidentially

Please complete the following document and return to the KWTRP CTF.

(please initial box to agree)

|  | I have received a copy of the IDMC Charter version 1 |
| --- | --- |
|  | I agree to attend the IDMC meeting on |
|  | I agree to treat as confidential any sensitive trial information gained during this meeting unless explicitly permitted |

Name: ___________________________

Signed: __________________________	Date: ______________

**Note:** This IDMC template was developed using MRC Clinical Trials Unit Cancer Group IDMC Charter template v2.01, 13-Mar-2006; from DAMOCLES IDMC Charter template v1, Feb 20
