# Supplementary material for: Gastroenteritis Aggressive Versus Slow Treatment For Rehydration (GASTRO). A pilot rehydration study for severe dehydration: WHO plan C versus slower rehydration
Source: Wellcome Open Res. 2017 Aug 10;2:62. [Version 1] doi: 10.12688/wellcomeopenres.12261.1 (PMC5571888; doi:10.12688/wellcomeopenres.12261.1)
Supplement: Peer review history

# Peer review: Referee response for version 1

**DOI:** 10.21956/wellcomeopenres.13274.r24938

**Published:** 2017-08-23

**Authors:** Melissa Parker

**Affiliations:** 1 Department of Pediatrics, McMaster University, Hamilton, ON, Canada

Thank you for the opportunity to review this interesting protocol manuscript.  Houston *et al. *present a well-written protocol manuscript describing a multi centre, unblinded Phase II pilot RCT of children 2 months to 12 years with severe acute gastroenteritis.  The two arms of the RCT consists of care per WHO Plan C (control) vs. slow rehydration (intervention).  

**Introduction**

 The rationale and need for the trial is well described.  The authors provide needed background with respect to existing practice and 'WHO plan C' for readers practicing in different clinical contexts.  Local audit data supports the contention that existing dogma and practice protocols should be challenged.  A systematic review has also recently been conducted.

**Methods**

 The trial methods are described in detail however some questions may remain for readers.  While the rehydration and resuscitative aspects of the intervention and control arm are clear, it is not entirely clear how the issue of maintenance fluids needs and ongoing excess losses are managed.  The authors do note that children are permitted to drink if able, or alternatively receive nasogastric fluid replacement, but it would be helpful those attempting to replicate the work to understand how maintenance fluid needs and replacement fluids (if applicable) for ongoing stool losses are being handled.  Is the approach protocolized within the trial or pragmatic?

 As noted by the authors, pilot trials are used to inform planning and for protocol refinement for a full-scale trial.  They are also used to inform feasibility.  The authors have selected reasonable study objectives considering that their protocol is challenging current practice.  On review of the statistical analysis plan (Supplementary File 3), the plan confirms that the primary outcome for the study is the frequency of predefined, fluid related, significant adverse events including; mortality, cardiovascular collapse, raised intracranial pressure, pulmonary oedema and allergic reaction.  This outcome will be analyzed as follows: The proportion of children with a pre-specified significant adverse event at 48hours in the Plan C and the GASTRO slow arm will be compared using an unadjusted chi-squared test on 1 degree of freedom Risk difference (95% CI) and risk ratio (95% CI) will also be presented.  What is interesting to note is that pilot trials are usually not powered to detect statistical significance (which the authors need not apologize for) and so it is unclear what the 'pass' threshold is for the authors in terms of numeric differences in adverse events between the control (WHO Plan C) and intervention arm.  The danger in employing statistical tests on the frequency of safety events in a small trial is that a) this provides false reassurance when real safety concerns may exist, or b) the authors are left in the uncomfortable position of finding a statistically significant difference in the setting of a small sample size (which may occur due to random chance) and which may threaten the ability to proceed with an otherwise worthwhile and important trial.  What is the authors pass threshold for moving on to the next stage?

**Ethics**

 Consent procedures are described in detail and this group has prior experience with deferred consent.  It is worth noting that failure to approach families of deceased children could result in differential drop-out between the two study arms and that data from these patients will not be included in the analysis.  This has potential implications for their primary (safety) outcome.  Other studies (in different clinical contexts) have approached decision-makers to seek consent following the death of a child and found this practice to be not only straightforward, but also of some potential benefit though such interactions are not intended to be therapeutic.

**Discussion**

 The authors present a compelling discussion and it is refreshing to see them share some preliminary feasibility information with respect to their both their struggles in implementing the pilot trial and their willingness to implement protocol changes when deemed necessary.  Research in the real world entails unanticipated challenges and the authors should be congratulated for their candor.

**Supplemental files**

 Useful information for readers is included as supplementary files, which are well organized and straightforward to follow.

**Additional**

 The manuscript contains some minor spelling and grammatical errors which are easily corrected, should post-publication corrections be allowed.

 Thank-you for the opportunity to review the interesting manuscript.  I look forward to learning more about this important trial.

I have read this submission. I believe that I have an appropriate level of expertise to confirm that it is of an acceptable scientific standard.

# Peer review: Referee response for version 1

**DOI:** 10.21956/wellcomeopenres.13274.r24935

**Published:** 2017-08-14

**Authors:** Srinivas Murthy

**Affiliations:** 1 Division of Critical Care, BC Children's Hospital, Vancouver, BC, V6H 3V4, Canada

This manuscript is a study protocol for an ongoing Phase II study of fluid resuscitation strategies in children with acute gastroenteritis and severe dehydration. Given the changing landscape of fluid administration strategies in severely ill children, this protocol describes a timely study comparing WHO-recommended resuscitation (100 ml/kg over 3-6 hours + boluses for shock) to a slower strategy (100 ml/kg over 8 hours without boluses for shock). They will be collecting in-depth physiologic data on a subset of children in both arms, including echocardiography, bio-impedance analysis, and microvascular perfusion analysis. 

 At time of writing, they are at nearly half of their target sample size, which was not formally determined, with primary objectives of safety, physiologic data, and an overall plan of informing a larger study with robust primary outcome measures. 

 This manuscript is well-written and convinces the reader as to the need for such a study to be completed. The protocol is clearly outlined, with clear descriptions of inclusion criteria and hoped-for management differences in intervention arms. The statistical analysis plan is included, forming the main impetus for publishing this protocol, and is organized well and addresses the major comparisons to be made, acknowledging the primary aims of this pilot study. 

 There are no specific amendments suggested to this publication.

I have read this submission. I believe that I have an appropriate level of expertise to confirm that it is of an acceptable scientific standard.
